# Arteriovenous Malformation of the Trapezius Muscle: A Report of a Rare Case

**DOI:** 10.7759/cureus.115022

**Published:** 2026-08-23

**Authors:** Amjad I Ilyas, Najla Nassar

**Affiliations:** 1 Department of Orthopedics, Security Forces Hospital, Makkah, SAU; 2 College of Medicine, King Saud bin Abdulaziz University for Health Sciences, Jeddah, SAU

**Keywords:** arteriovenous malformation, embolization, intramuscular vascular malformation, sclerotherapy, trapezius muscle, vascular anomaly

## Abstract

Arteriovenous malformations (AVMs) are rare congenital fast-flow vascular anomalies characterized by direct arteriovenous shunting through a nidus. Intramuscular AVMs are uncommon and are frequently mistaken for intramuscular hemangiomas or venous malformations, and involvement of the trapezius muscle is exceptionally rare. We report the case of a 31-year-old woman with a five-year history of chronic left-sided cervical pain that had been managed as a muscle spasm with no improvement. Examination revealed a fixed trapezius mass with localized tenderness. Ultrasonography and magnetic resonance imaging (MRI) demonstrated an intramuscular tangle of dilated vessels, and catheter angiography confirmed a high-flow AVM. Following multidisciplinary discussion, the patient underwent transarterial embolization and sclerotherapy with near-complete obliteration of the shunt and remained symptom-free without recurrence at two years. AVMs are suggested to be considered in persistent, atypical neck and shoulder masses, where minimally invasive endovascular treatment can yield excellent outcomes.

## Introduction

Vascular anomalies are broadly divided into vascular tumors, which arise from endothelial proliferation, and vascular malformations, which are structural errors of vascular morphogenesis without true proliferation [1,2]. Within the framework of the International Society for the Study of Vascular Anomalies (ISSVA), malformations are further subclassified by their predominant channel type and hemodynamic behavior into slow-flow lesions (capillary, venous, and lymphatic malformations) and fast-flow lesions, of which the arteriovenous malformation (AVM) is the principal example [1,2]. This distinction is clinically decisive, because flow characteristics determine the imaging strategy, the therapeutic approach, and the likelihood of recurrence [3,4].

AVMs are congenital, high-flow lesions characterized by a nidus of dysplastic arteriovenous connections that shunt blood directly from the arterial to the venous circulation, bypassing the capillary bed [1,4]. Although present at birth, they may remain quiescent for years before becoming clinically apparent [4]. Sustained high-flow shunting drives progressive dilatation and remodeling of the feeding arteries and draining veins, venous hypertension, and vascular “steal” from adjacent tissues, producing a spectrum of symptoms that ranges from pain and swelling to ulceration, hemorrhage, neurological compromise, and, in extensive lesions, high-output cardiac failure [4].

Intramuscular vascular malformations are uncommon and are frequently mislabeled as “intramuscular hemangiomas," a term that erroneously conflates proliferative tumors with malformations and contributes to diagnostic confusion and inappropriate treatment [5]. When they arise within skeletal muscle, they most commonly involve the muscles of the lower extremity, particularly the thigh and calf, whereas the shoulder girdle and neck are affected far less often [5,6]. Fast-flow AVMs are rarer than their slow-flow venous counterparts in this setting, and their deep, ill-defined presentation with activity-related pain readily mimics ordinary musculoskeletal disease [5,6].

AVMs arising specifically within the trapezius muscle are exceptionally rare, and reports of high-flow lesions in the shoulder girdle are few [7]. Because the trapezius is a large, superficial muscle whose discomfort is routinely attributed to strain, cervicalgia, or myofascial spasm, diagnostic anchoring to these common conditions can delay recognition for years, allow the lesion to progress, and expose the patient to repeated ineffective interventions [5,7]. We report a rare case of an intramuscular AVM of the trapezius muscle that was successfully diagnosed and treated through a minimally invasive approach.

## Case presentation

Clinical presentation and history

A 31-year-old woman with no known chronic medical illness presented to the orthopedic clinic in early November 2021 with a five-year history of left-sided cervical pain. The pain was non-radiating and was described as a dull sensation of fullness, without associated numbness or weakness. It began gradually and increased over time, becoming markedly more intense during the preceding five months. Symptoms were aggravated by physical activity and prolonged working hours and disturbed her sleep, with only partial relief from rest and analgesics. She had previously been assessed by another physician, diagnosed with a simple muscle spasm, and treated conservatively with medications and physiotherapy, without achieving the intended improvement. Her past medical and surgical history was unremarkable, with no history of trauma or preceding injury. She was a current smoker and reported no known drug allergies.

Examination

On examination, she was of average build and demonstrated generalized ligamentous laxity with a Beighton score of 6/9 [8]. Inspection of the neck revealed no significant deformity or scar. There was moderate soft-tissue swelling on the left side of the neck with a fixed mass measuring approximately 5 × 3 cm at the posterosuperior aspect of the left shoulder, without overlying warmth or erythema. Localized tenderness was elicited over the posterosuperior left shoulder at the level of the trapezius muscle, with no other site of tenderness in the neck or shoulder. The range of motion of the neck and shoulder was normal, the left shoulder was stable, and the distal neurovascular examination was unremarkable.

Investigations and procedure

**Figure 1:**
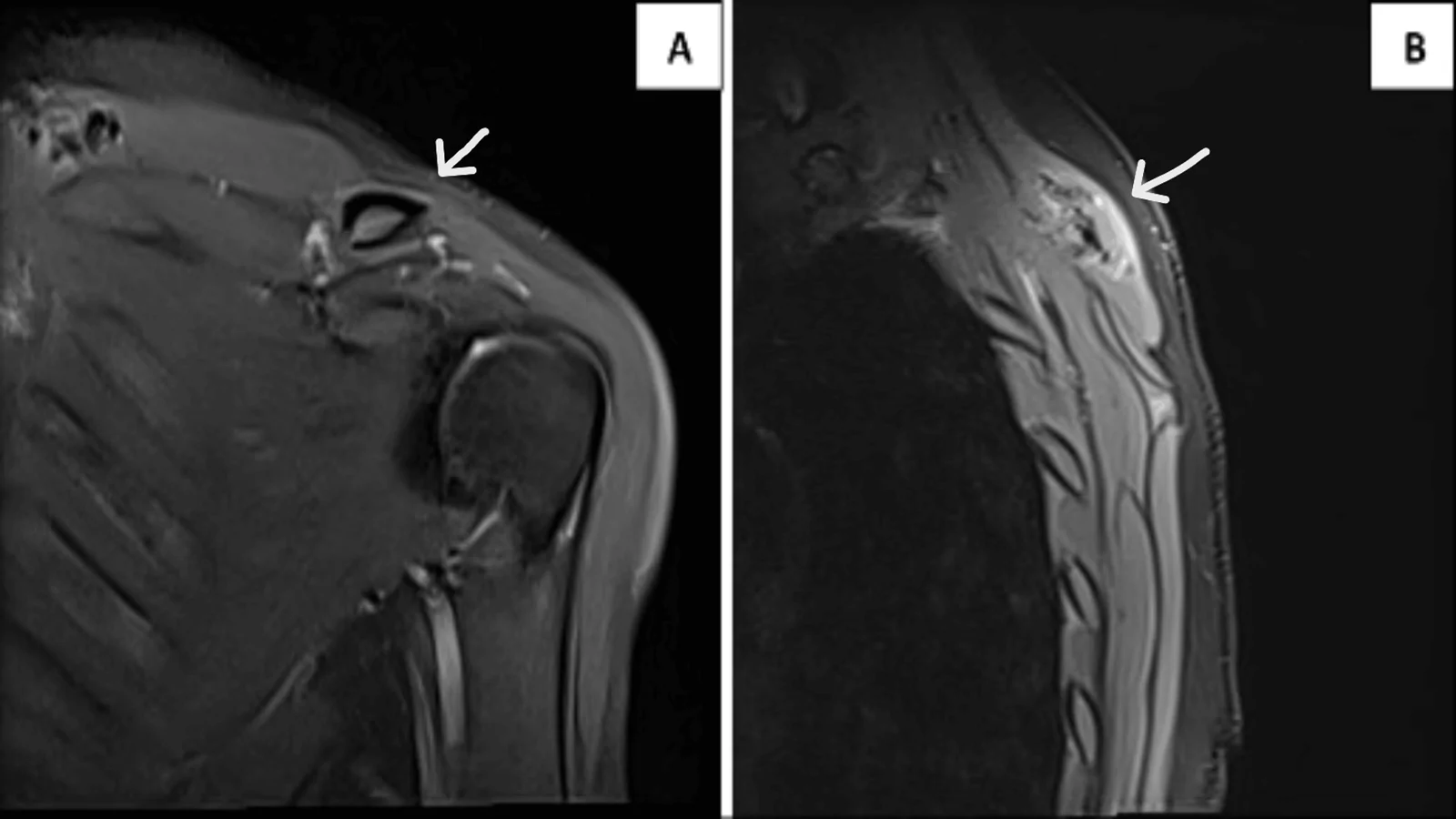
Magnetic resonance imaging of the left trapezius region. (A) Sagittal image showing an ill-defined intramuscular lesion within the left trapezius. (B) Coronal image demonstrating a tangle of serpentine, dilated vessels (flow voids) consistent with a high-flow intramuscular vascular malformation.

**Figure 2:**
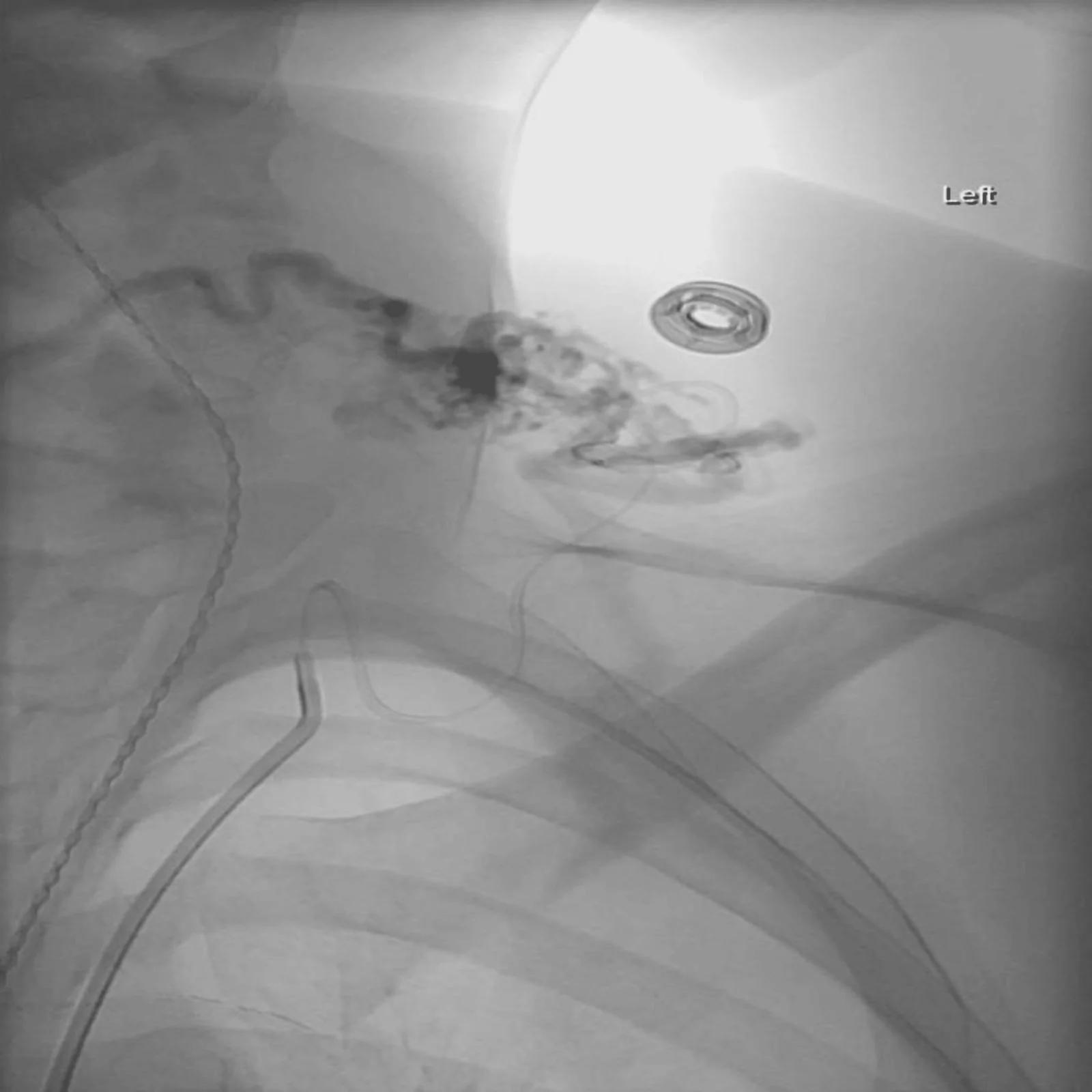
Intraprocedural angiogram of the left trapezius arteriovenous malformation showing the nidus and its arterial feeder.

Plain radiographs of the cervical spine and the left shoulder were unremarkable. Ultrasonography of the left neck and shoulder swelling demonstrated ill-defined muscle echogenicity with fatty infiltration and features suggesting an intramuscular soft-tissue abnormality, prompting further characterization with magnetic resonance imaging (MRI). MRI revealed an intramuscular lesion within the left trapezius measuring approximately 3.6 × 2.1 × 4 cm, comprising a tangle of serpentine, dilated vessels consistent with an underlying high-flow vascular malformation (Figures 1A, 1B). Catheter-directed angiography subsequently confirmed a fast-flow AVM and identified the arterial feeder supplying the nidus (Figure 2).

Differential diagnosis

On the basis of a fixed, tender intramuscular mass with imaging evidence of a vascular lesion, the differential diagnosis included intramuscular hemangioma, venous malformation, lymphatic malformation, soft-tissue sarcoma, and pseudoaneurysm.

Treatment and outcome

After discussion of the imaging findings, the patient initially agreed to continue non-operative management with analgesia and physical therapy, alongside referral to vascular surgery and interventional radiology. Catheter-directed angiography subsequently confirmed a fast-flow AVM feeder supplying the nidus, which was documented intraprocedurally (Figure 2). The demonstration of arteriovenous shunting on angiography established the diagnosis of an AVM and excluded the slow-flow malformations and non-vascular soft-tissue tumors. Following multidisciplinary team review, a trial of endovascular embolization and sclerotherapy was planned and performed by interventional radiology in late November 2022. The feeding artery, a muscular branch of the transverse cervical artery, was selectively catheterized, and embolization of the malformation was carried out under image guidance with adjunctive sclerotherapy of the lesion. The nidus was devascularized with preservation of the adjacent normal vasculature, and the procedure was well tolerated without immediate complications. The post-procedural angiogram demonstrated near-complete obliteration of the arteriovenous shunt.

Post-procedural management and follow-up

The patient recovered well and was discharged on the same day. Routine outpatient follow-up showed significant improvement progressing to complete symptom resolution, and at two years after treatment, there has been no clinical recurrence. 

## Discussion

AVMs are congenital, high-flow lesions in which a nidus of dysplastic channels allows direct high-pressure arterial flow into the venous system without an intervening capillary bed [1,4]. Their designation as fast-flow lesions within the ISSVA classification is clinically important since it separates them from slow-flow venous and lymphatic malformations and dictates a fundamentally different imaging and treatment strategy [1,2]. Intramuscular vascular malformations most often involve the lower limb, and trapezius involvement is exceptionally rare [5,6]; the broad, superficial anatomy of this muscle means that its pain is habitually ascribed to strain or myofascial spasm, precisely the initial diagnosis in our patient, which contributed to a five-year delay before the lesion was correctly identified [7].

Our findings align with the limited published experience of high-flow lesions in the shoulder girdle. Cusimano and colleagues reported multiple AVMs surrounding the shoulder joint, with pain localized in part to the trapezius, that were successfully managed by transarterial and direct-puncture embolization; as in our case, the diagnosis had been obscured by an initial assumption of musculoskeletal injury, and endovascular therapy produced substantial symptomatic relief [7]. A series of intramuscular malformations elsewhere in the body similarly emphasizes both the frequency of misdiagnosis and the favorable response to targeted, minimally invasive treatment [5,6]. Our case extends this experience by documenting a discrete AVM confined to the trapezius that was durably controlled by embolization and sclerotherapy.

Imaging is central to diagnosis and to distinguishing an AVM from its mimics. Doppler ultrasonography is a useful first-line study that can reveal high-velocity, low-resistance arterial flow suggestive of shunting, while MRI best delineates lesion extent and typically shows serpentine vessels and flow voids in high-flow lesions [2,3]. Catheter angiography remains the reference standard, uniquely able to demonstrate the nidus, characterize flow dynamics, and serve as the platform for treatment in the same setting [4,9]. These modalities reliably separate an AVM from an intramuscular hemangioma, which is a true proliferative tumor; from venous and lymphatic malformations, which are slow-flow lesions lacking arteriovenous shunting; from a soft-tissue sarcoma, which is an infiltrative solid mass; and from a pseudoaneurysm, which is a contained arterial disruption communicating with a single vessel rather than a nidus [2,6].

Management is directed at symptom control rather than guaranteed cure, since complete eradication is often unattainable in diffuse lesions [4]. Endovascular embolization, increasingly regarded as first-line therapy for symptomatic AVMs, occludes the nidus and feeding arteries and is particularly advantageous for deep-seated lesions because it minimizes surgical trauma; it is frequently combined with sclerotherapy [4,7,10]. Surgical excision can be curative for small, well-circumscribed, superficial lesions but carries a substantial risk of hemorrhage and incomplete resection when lesions are large or diffuse and is therefore often reserved for combined therapy after preoperative embolization [5,6].

Because incomplete treatment or proximal arterial ligation can provoke collateral recruitment and recurrence, targeted nidus obliteration and long-term follow-up with interval imaging are essential [4,8]. In our patient, near-complete angiographic obliteration was achieved by embolization and sclerotherapy, with sustained complete recovery at two years, an outcome consistent with the better results reported for focal, accessible lesions [7,10,11].

## Conclusions

AVM of the trapezius muscle is an exceptionally rare cause of chronic neck and shoulder pain that is easily mistaken for benign musculoskeletal disease, leading to diagnostic delay. Clinicians should maintain a low threshold for Doppler and cross-sectional imaging in patients with persistent, atypical, or treatment-resistant neck or shoulder masses, and should include vascular malformations in the differential diagnosis. In this patient, early recognition combined with a multidisciplinary, minimally invasive endovascular approach achieved complete symptom resolution with no recurrence at two years, although outcomes from a single case may not be generalizable to all trapezius AVMs.
